# Dysbindin deficiency Alters Cardiac BLOC-1 Complex and Myozap Levels in Mice

**DOI:** 10.3390/cells9112390

**Published:** 2020-10-31

**Authors:** Ankush Borlepawar, Nesrin Schmiedel, Matthias Eden, Lynn Christen, Alexandra Rosskopf, Derk Frank, Renate Lüllmann-Rauch, Norbert Frey, Ashraf Yusuf Rangrez

**Affiliations:** 1 Department of Internal Medicine III (Cardiology, Angiology, Intensive Care), University Medical Center Kiel, 24105 Kiel, Germany; ankush.borlepawar@uksh.de (A.B.); nesrin.schmiedel@uksh.de (N.S.); matthias.eden@uksh.de (M.E.); lynn.christen@web.de (L.C.); alexandra.rosskopf@uksh.de (A.R.); derk.frank@uksh.de (D.F.); norbert.frey@uksh.de (N.F.); 2 DZHK (German Centre for Cardiovascular Research), Partner Site Hamburg/Kiel/Lübeck, 24105 Kiel, Germany; 3 Institute of Anatomy, Christian-Albrechts-University Kiel, 24118 Kiel, Germany; r.lullmann@anat.uni-kiel.de

**Keywords:** Dysbindin, cardiac hypertrophy, Myozap, Pallidin, Muted

## Abstract

Dysbindin, a schizophrenia susceptibility marker and an essential constituent of BLOC-1 (biogenesis of lysosome-related organelles complex-1), has recently been associated with cardiomyocyte hypertrophy through the activation of Myozap-RhoA-mediated SRF signaling. We employed sandy mice (*Dtnbp1*_KO), which completely lack Dysbindin protein because of a spontaneous deletion of introns 5–7 of the *Dtnbp1* gene, for pathophysiological characterization of the heart. Unlike in vitro, the loss-of-function of Dysbindin did not attenuate cardiac hypertrophy, either in response to transverse aortic constriction stress or upon phenylephrine treatment. Interestingly, however, the levels of hypertrophy-inducing interaction partner Myozap as well as the BLOC-1 partners of Dysbindin like Muted and Pallidin were dramatically reduced in *Dtnbp1*_KO mouse hearts. Taken together, our data suggest that Dysbindin’s role in cardiomyocyte hypertrophy is redundant in vivo, yet essential to maintain the stability of its direct interaction partners like Myozap, Pallidin and Muted.

## 1. Introduction

Cardiac hypertrophy may be reversible and beneficial, adapting according to systemic demands such as exercise or pregnancy. In contrast, its maladaptive form, termed “pathological hypertrophy”, is associated with maladaptive molecular changes ranging from epigenetic to translational level that eventually may lead to the heart failure [1,2]. Pathological hypertrophy induces cardiac remodeling including interstitial fibrosis, capillary rarefaction, increased production of pro-inflammatory cytokines, and cardiomyocyte dysfunction leading to advanced cardiomyopathy [3,4]. Among the multiple signaling pathways investigated over the last decades that are involved in cardiomyocyte hypertrophy and cardiomyopathy pathogenesis, induction of the cardiomyocyte hypertrophic gene program via the master transcription factor-serum response factor (SRF) is of crucial importance [5,6,7]. A cardiac enriched intercalated disc (ID) protein Myozap was reported to be a strong inducer of SRF-mediated cardiac hypertrophy [8]. Over time, our group revealed several other key players like Dysbindin, GTPases like RhoA/Rnd1, and TRIM family members like TRIM24/TRIM32, which lead to either induction or inhibition of SRF-signaling mediated cellular hypertrophy in neonatal rat cardiomyocytes. We initially discovered Dysbindin via a yeast two-hybrid screen of Myozap against human cardiac cDNA library that we later found to be interacting with almost all these proteins [9,10,11], suggesting the involvement of an orchestrated, multi-protein complex in the induction of SRF-mediated cardiomyocyte hypertrophy.

Dysbindin, which is expressed in several tissues including the heart [11], is part of the ubiquitously expressed BLOC-1 complex (biogenesis of lysosome-related organelles complex 1), also consisting of proteins Pallidin, Snapin, Cappucino, Muted, along with 1-3 BLOS proteins [12]. This octet is essential for the normal biogenesis of various organelles that are part of the endosomal–lysosomal system, protein sorting along with complex -2/3, membrane biogenesis and vesicular trafficking [13,14,15]. Two stable sub-complexes were furthermore defined as trimers of Pallidin-Cappuccino-BLOS1 and Dysbindin-Snapin-BLOS2 [16]. The defects in the proteins belonging to the BLOC-1 complex have been credited with numerous diseases like variations of Hermansky–Pudlak syndrome (HPS) [17]. Moreover, Dysbindin has previously been a well-studied protein in the neuronal context, where it plays a central role in neurite outgrowth and cargo trafficking via both pre- and post-synaptic neurons [18,19,20]. Reduced Dysbindin protein levels in the brain eventually promote to schizophrenia, establishing Dysbindin as a prominent Schizophrenia susceptibility protein [18,21,22].

With such a widely studied role in neuronal pathophysiology, we recently also performed a detailed characterization of Dysbindin in neonatal rat cardiomyocytes, where it was found to play a distinct role in the induction of hypertrophic cardiac signaling, in particular, RhoA–SRF axis-dependent transcription [11]. In the current manuscript, we used Dysbindin-deficient mice to study cardiac (patho)physiology at baseline and under stress conditions like TAC and PE treatment. Surprisingly, the loss-of-function of Dysbindin in vivo did not attenuate cardiac hypertrophy induced by TAC/PE infusion. Interestingly, however, the levels of hypertrophy-inducing interaction partner Myozap and BLOC-1 partners of Dysbindin like Muted and Pallidin were dramatically reduced in *Dtnbp1*_KO mouse heart. Taken together, these data suggest that Dysbindin likely plays an important role in maintaining the integrity of its interaction partners like Myozap, Pallidin and Muted in the heart.

## 2. Materials and Methods

### 2.1. Experimental Animals

All animal experiments were performed in male *Dtnbp1*_KO mice in C57BL/6J genetic background [23] in accordance with the institutional guidelines of the Kiel University and regulations of the local ethical committee (Ministerium für Energiewende, Landwirtschaft, Umwelt und Ländliche Räume Schleswig-Holstein (MELUND)) of the state Schleswig-Holstein, Germany. All the animal experimental protocols were approved by the ethical committee at MELUND (reference number—V242-7224.121-4(72-6/14)). Tail snips from all animals were genotyped using a duplex PCR procedure yielding PCR products across the deleted *Dtnbp1* segment in *Dtnbp1*_KO mice.

### 2.2. Genotyping

To confirm the absence of Dysbindin in the Sandy mice, we performed genotyping PCR on DNA obtained from tail-clippings of mice. The primer composition consisted of a mixture of four different primers specific for Wild-type (Wild-type_Fw: 5′-ATA CCG GAG ATC ATG CAA GC-3′, _Rv: 5′-AGC TCC ACC TGC TGA ACA TT-3′) and *Dtnbp1*_KO (*Dtnbp1*_KO_Fw: 5′-TCC TTG CTT CGT TCT CTG CT-3′, _Rv: 5′-CTT GCC AGC CTT CGT ATT GT-3′) mice. The PCR conditions applied for genotyping were 3 min at 94 °C for primary denaturation, followed by 31 cycles of 30 s at 94 °C for denaturation, 45 sec at 56 °C for annealing and 1 min at 72 °C for the extension. The product lengths for Dysbindin achieved after loading the PCR product on agarose gel were 472 bps in Wild-type and 274 bps in *Dtnbp1*_KO mice, confirming the knock-out of the gene (Figure S1A).

### 2.3. Antibodies

The antibodies used for various immunoblotting experiments in this study were as follows: GAPDH, mouse monoclonal (1:20,000; Sigma, St. Louis, MO, USA); Muted, rabbit polyclonal (1:1000, Proteintech, Manchester UK); Myozap, mouse monoclonal (1:150; Progen, Heidelberg, Germany); Pallidin, rabbit polyclonal (1:1000, Proteintech); Rnd1, rabbit polyclonal (1:1000, LSBio via Biozol, Seattle, WA, USA); SERCA2A, mouse monoclonal (1:1000, Thermo Scientific, Waltham, MA, USA); α-Tubulin, mouse monoclonal (1:8000, Sigma).

### 2.4. Protein Isolation and Western Blotting

Protein extraction and immunoblotting was performed as previously described [9]. Briefly, mouse hearts were homogenized using a Precellys homogenizer with coarse and fine plastic beads in RIPA buffer (1.0 mM Tris, 5 mM EDTA, pH 7.5, 1% NP-40 (*v*/*v*), 0.5% sodium deoxycholate (*w*/*v*), 0.1% SDS (*w*/*v*)) supplemented with protease inhibitor cocktail (Roche Applied Science, Penzberg, Germany) and phosphatase inhibitors 2 and 3 (Sigma). Protein concentration was determined by the DC assay kit according to the manufacturers’ instructions (Bio-Rad, Hercules, CA, USA). Protein samples prepared with Laemmli buffer were resolved by SDS-PAGE with a 10% polyacrylamide gel and transferred to nitrocellulose membranes. Membranes were then incubated overnight at 4 °C with target-specific primary antibodies diluted in 5% milk/BSA prepared in TBST buffer. Subsequently, the incubation in the HRP-coupled secondary antibody was carried out for 1 h at room temperature. Proteins were visualized with the help of a GelDoc using the chemiluminescence kit (ECL-select; GE Healthcare, Chicago, IL, USA) and bands were detected by the FluorChem Q imaging system (Biozym, Hessisch Oldendorf, Germany). Densitometry analyses were performed with the ImageJ software version 1.46 by measuring specific proteins against the cellular housekeeping proteins like Tubulin or GAPDH for normalization.

### 2.5. RNA Isolation and qRT-PCR

Total RNA was isolated from mice hearts using TRIzol-based QIAzol lysis reagent (Qiagen, Hilden, Germany) and a Precellys homogenizer with coarse and fine plastic beads. Reverse transcription into cDNA was carried out from 1 µg of DNA-free RNA using the Superscript III first-strand cDNA synthesis kit (Life Technologies, Inc., Carlsbad, CA, USA). A CFX96 real-time cycler (Bio-Rad) was used for performing the PCR-based amplification using EXPRESS SYBR Green ER reagent (Life Technologies, Inc.) in the qRT-PCR setup. Cycling conditions used were as follows: 3 min at 95 °C for denaturation, followed by 40 cycles of 15 s at 95 °C for annealing and 45 s at 60 °C for the extension. Housekeeping gene *Rpl32* was used for the normalization.

### 2.6. Transverse Aortic Constriction, Phenylephrine (PE) Osmotic Pump Implantation and Echocardiography

TAC and echocardiography were performed in 8-week-old male *Dtnbp1*_KO mice and their wild-type counterparts as described previously [9,24]. Briefly, mice were anesthetized with a combination of ketamine (120 mg/kg i.p.) and xylazine (15 mg/kg i.p.). Mice were then orally intubated with a 20-gauge tube and ventilated (Harvard Apparatus, Holliston, MA, USA) at 120 breaths per min (0.2 mL tidal volume). The aortic constriction was performed via a lateral thoracotomy through the second intercostals space. A suture (Prolene 6-0) was placed around the transverse aorta between the brachiocephalic and left carotid artery and ligated against a 27-gauge needle. The needle was later removed leaving discrete stenosis, the chest was sutured and the pneumothorax evacuated. Sham-operated animals underwent the same procedure except for ligation. For PE treatment, osmotic mini-pumps filled with Phenylephrine (20 µg/kg body weight/min) prepared in PBS with 1mg/mL L-ascorbate (Sigma) were implanted subcutaneously [9]. The Control group received vehicle L-ascorbate in PBS. Cardiac function of experimental animals was examined by echocardiography 2 weeks post-surgery or -minipump implantation before sacrificing mice to collect heart for downstream applications.

### 2.7. Electron Microscopy

Ultrastructure of *Dtnbp1*_KO mice was observed by electron microscopy as previously described [24]. In brief, *Dtnbp1*_KO mice were weighed and anesthetized with an intraperitoneal injection consisting of ketamine (12 mg/mL) and xylazine (1.6 mg/mL) (10 µL/g body weight). The heart was pre‑perfused with 1% procaine in 0.1 M PBS and fixed with 6% glutaraldehyde in 0.1 M PBS by transcardial vascular perfusion before harvest papillary muscles for further examination. Tissue blocks were post-fixed with 2% osmium tetroxide and embedded in Araldite. Ultra-thin sections were processed with uranyl acetate and lead citrate and viewed with Zeiss EM900 microscope (Carl Zeiss, Jena, Germany).

### 2.8. Statistical Analyses

All the presented results are the means ± S.E. unless stated otherwise. Statistical analyses were performed using a two-tailed Student’s t-test or two-way ANOVA (followed by Student–Newman–Keuls post-hoc tests when appropriate), respectively. *P* values ≤ 0.05 were considered statistically significant.

## 3. Results

### 3.1. Dysbindin-Deficient Mice do not Exhibit Altered Cardiac Phenotype at Baseline

**Figure 1:**
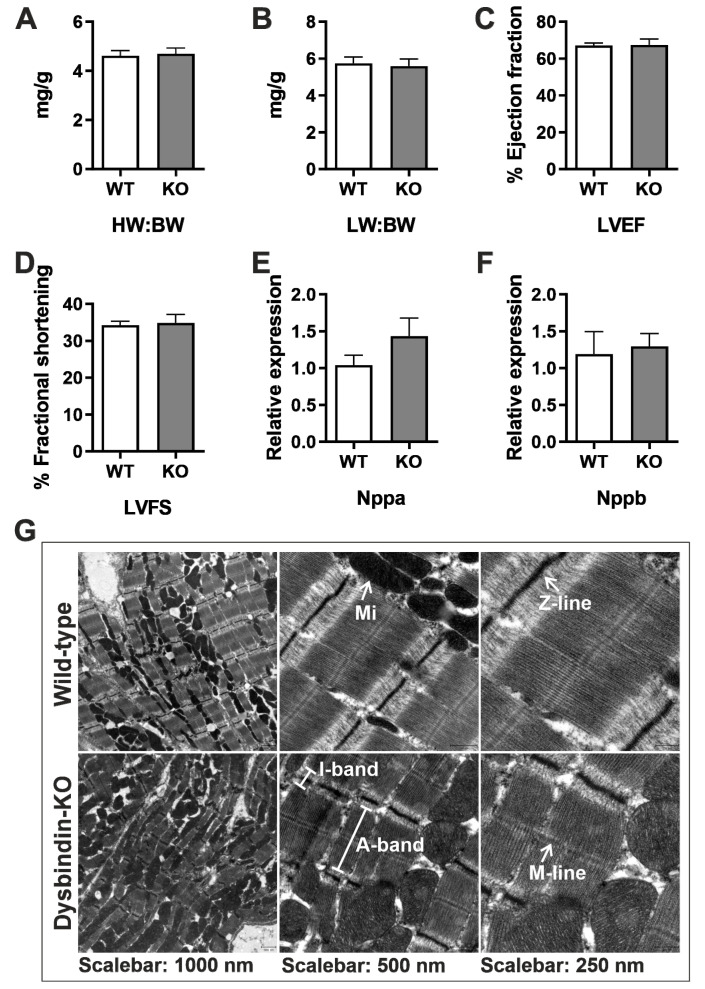
Basal characterization of *Dtnbp1*_KO mice. Morphometric characterization comparing parameters of 3-month-old *Dtnbp1*_KO and wild-type mice (n = 6 each) for heart wt.: body wt. (**A**), lung wt.: body wt. (**B**), percentage of left ventricle ejection fraction (**C**), percentage of left ventricle fractional shortening (**D**). Expression of hypertrophic genes *Nppa* (**E**) and *Nppb* (**F**) was determined by quantitative real-time PCR. (**G**) Electron microscopic images at various optical magnifications showing the architecture of cardiac muscle. ID: intercalated disc; Mi: mitochondria. Statistical significance was calculated by Student’s t-test. Error bars show mean ± S.E.

We previously reported Dysbindin as a robust inducer of cellular hypertrophy via induction of RhoA-mediated SRF signaling in neonatal rat cardiomyocytes [11]. To gain a deeper understanding of the cardiac function of Dysbindin, we used Dysbindin-deficient ‘*sandy*’ mice (*Dtnbp1*_KO, Figure S1A,B). *Sandy* is an autosomal recessive coat mutation that spontaneously occurred in 1983 at The Jackson Laboratory in the inbred DBA/2J strain [23]. For this study, however, the mutation was transferred to the C57BL/6J (B6) genetic background by 11 generations of backcrossing into B6 to remove the strong abnormal behavioral, mental and locomotor phenotypes in DBA/2J compared to B6 background [23], which we believed might alter cardiac function. We characterized these mice at the age of 12 weeks and 1 year for the cardiac phenotype in unstressed mice. The phenotypic and morphometric characteristics observed by ratios of heart weight and lung weight against the bodyweight, and functional observations stating percentages of left ventricular ejection fraction and fractional shortening by echocardiography in 12 weeks old mice did not project any hypertrophy-related characteristics (Figure 1A–D). Similarly, there was no effect of Dysbindin deficiency on hypertrophic gene program as assessed by the expressions of natriuretic peptides *Nppa* and *Nppb* and some of the known SRF gene targets (Figure 1E,F and Figure S1C). Moreover, electron microscopy-based myocardial investigation revealed no obvious ultrastructural abnormalities in the *Dtnbp1*_KO mouse heart compared to wild-type littermates (Figure 1G). Cardiac function in 1-year-old *Dtnbp1*_KO mice was indifferent from wild-type littermates (data not shown). These data thus suggest no deleterious effects of Dysbindin deficiency on cardiac structure and physiological function at baseline.

### 3.2. Dysbindin Deficiency Does not Alter Cardiac Hypertrophy Due to Pressure Overload

**Figure 2:**
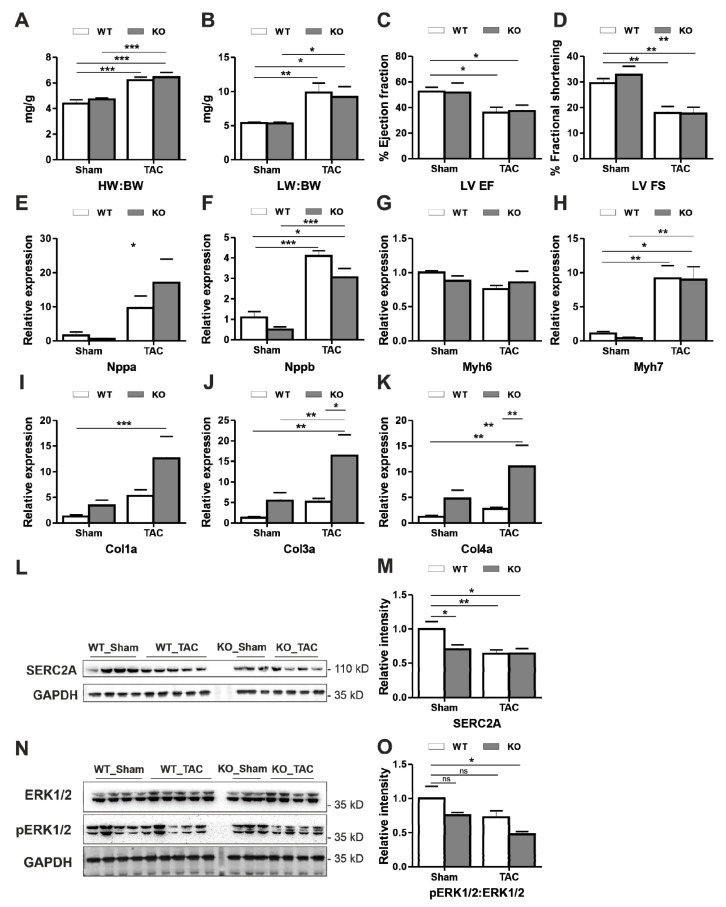
Characterization of *Dtnbp1*_KO mice in biomechanical stress-induced cardiomyopathy. TAC or sham operations were performed on 8-week old wild-type (WT) and *Dtnbp1*_KO mice. Post two weeks of operations (n = 7 (WT-SHAM), 8 (WT-TAC), 8 (KO-SHAM), 8 (KO-TAC)). Morphometric characterization showing ratios of heart weight (wt):body wt (**A**) and lung wt:body wt (**B**), functional characterization using the percentage of left ventricle ejection fraction (**C**) and percentage of left ventricle fractional shortening (**D**). Expression of hypertrophic genes *Nppa* (**E**), *Nppb* (**F**), myosin heavy chain (*Myh7*) (**G**), myosin light chain (*Myh6*) (**H**) and fibrotic markers *Col1a* (**I**), *Col3a* (**J**) and *Col4a* (**K**) was determined by quantitative real-time PCR. Representative Immunoblots display cardiac levels of SERC2A (**L**), with its densitometry analysis in (**M**). Representative Immunoblots display cardiac levels of ERK1/2 and pERK1/2 (**N**), with its densitometry analysis in (**O**). Statistical significance was calculated by two-way ANOVA. Error bars show mean ± S.E. ns, non-significant; *, *p* < 0.05; **, *p* < 0.01; ***, *p <* 0.001.

Since Dysbindin is significantly upregulated in mouse models of pressure overload due to TAC or PE treatment (Figure S2A,B), despite no cardiac abnormalities in *Dtnbp1*_KO mice at the baseline, we aimed to determine whether Dysbindin is necessary for cardiac adaptation against cardiac pressure overload due to TAC. At first, no significant differences were observed in the survival of *Dtnbp1*_KO and wild-type mice after TAC (Figure S2C). The phenotypic characteristics measured by parameters like heart:body (Figure 2A) or lung:body (Figure 2B) weight ratios, left ventricular functions like ejection fraction (Figure 2C) and fractional shortening (Figure 2D) strongly portrayed the hypertrophic condition of heart upon due to pressure overload. The TAC operations also induced expected hypertrophy at the molecular level as evidenced from increased expression of markers like natriuretic peptides *Nppa* (Figure 2E), *Nppb* (Figure 2F), myosin heavy chain-7 (*myh7*) (Figure 2H), and downregulation of *myh6* (Figure 2G). Notwithstanding, the lack of Dysbindin did not alter the pathological outcome of the heart after TAC (Figure 2A–H). Interestingly, though, the expression of collagens, fibrosis markers, was significantly higher in *Dtnbp1*_KO than respective control mice (Figure 2I–K). Similarly, Dysbindin deficiency strongly reduced protein levels of SERC2A not only after TAC but also in sham-operated mice (Figure 2L, M). Interestingly, however, activation of ERK1/2 was significantly attenuated in *Dtnbp1*_KO mice after TAC, whereas only a trend of downregulation of pERK1/2 was observed in wild-type littermates (Figure 2N,O).

### 3.3. Dysbindin Deficiency Does not Alter Cardiac Hypertrophy Due to Phenylephrine (PE) Treatment

**Figure 3:**
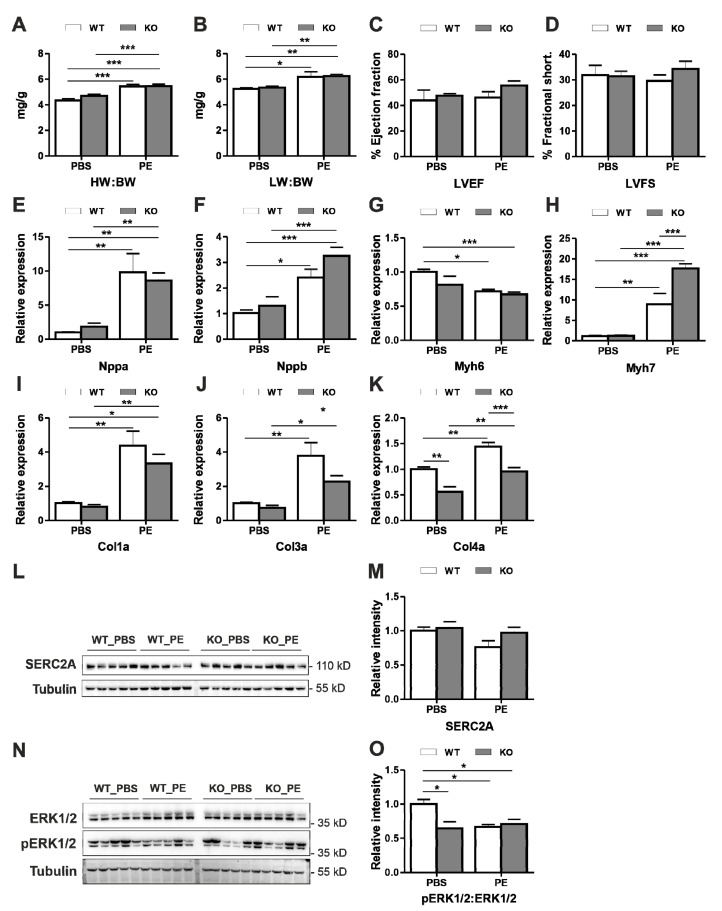
Characterization of *Dtnbp1*_KO mice in pharmacologically induced alpha-adrenergic stimulation -induced cardiomyopathy. 8-week old wild-type (WT) and *Dtnbp1*_KO mice underwent PE or PBS (control) introduction using osmotic minipumps implantation. Post two weeks of implant (*n* = 4 (WT-PBS), 5 (WT- PE), 5 (KO- PBS), 10 (KO- PE)), phenotypic characterization was performed by measuring morphometric characters, ratios of heart weight (wt): body wt (**A**), lung wt: body wt (**B**), functional characters like percentage of left ventricle ejection fraction (**C**) and percentage of left ventricle fractional shortening (**D**). Expression of hypertrophic genes *Nppa* (**E**), *Nppb* (**F**), myosin heavy chain (**G**), myosin light chain (**H**) and fibrotic markers *Col1a* (**I**), *Col3a* (**J**) and *Col4a* (**K**) was determined by quantitative real-time PCR. Representative Immunoblots display cellular levels of SERC2A (**L**), with its densitometry analysis in (**M**). Representative Immunoblots display cardiac levels of ERK1/2 and pERK1/2 (**N**), with its densitometry analysis in (**O**). Statistical significance was calculated by two-way ANOVA. *Error bars* show mean ± S.E. *, *p* < 0.05; **, *p* < 0.01; ***, *p <* 0.001.

Earlier, we observed a strong inhibition of cellular hypertrophy induced by PE when Dysbindin was knocked down in NRVCMs [11]. To determine if similar inhibitory action translates in vivo, we treated mice with PE using a subcutaneous introduction of osmotic mini-pumps. Control mice received PBS. As anticipated, PE induced the alpha-adrenergic stimulation hypertrophic signature in the mice hearts, with upregulation of phenotypic, functional and molecular markers mentioned earlier (Figure 3A–H). However, again much against anticipation, there was no significant difference between *Dtnbp1*_KO and wild-type PE treated mice in most of the parameters except for significant upregulation of Myh7 and significant downregulation of collagens (Figure 3H,J,K). Change in the hypertrophic signature was observed in accordance with the absence of Dysbindin, strongly suggesting non-translation of in vitro effects. The majority of the parameters, like heart/body (Figure 3A) or lung/body (Figure 3B) weights, left ventricular functions like ejection fraction (Figure 3C) and fractional shortening (Figure 3D), or the expression of hypertrophic genes (Figure 3E–H) in PE-treated *Dtnbp1*_KO mice were not indifferent from the respective wild-type groups. The vasopressor like PE is also known to induce the fibrotic markers like Col1a, Col3a and Col4a; interestingly, however, Col3a and Col4a were markedly downregulated by the absence of Dysbindin (Figure 3I–K) after PE-induced hypertrophy, implying a possibly beneficial effect of Dysbindin deficiency. Moreover, SERCA2A (Figure 3L,M), which was dysregulated after TAC operations in *Dtnbp1*_KO mice, was also found unaltered after PE treatment. Moreover, activation of ERK1/2 was robustly reduced in PE treated mice of both genotypes, this effect was however persistent in PBS treated *Dtnbp1*_KO mice as well (Figure 3N,O).

### 3.4. Myozap and BLOC-1 Complex Are Dysregulated after Knock-Out of Dysbindin

**Figure 4:**
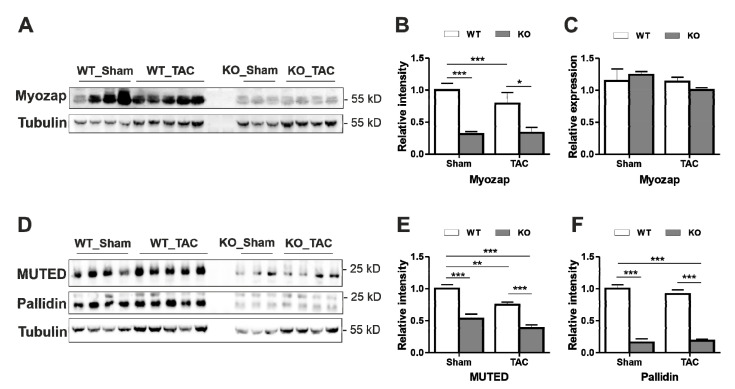
Myozap and BLOC-1 complex are dysregulated after knock-out of Dysbindin. (**A**) Immunoblots depicting Myozap protein levels in Sham/TAC operated mice, its densitometric analysis against Tubulin shown in (**B**) and transcript levels in (**C**). (**D**) Immunoblots depicting the protein levels of BLOC-1 components Muted and Pallidin along with their densitometric analysis against Tubulin shown in (**E**) and (**F**), respectively. Statistical significance was calculated by two-way ANOVA. Error bars show mean ± S.E. *, *p* < 0.05; **, *p* < 0.01; ***, *p <* 0.001.

To find the potential molecular causes of inconsistent in vitro and in vivo observations in the role of Dysbindin in cardiomyocyte hypertrophy, we determined the expression levels of some of the known interaction partners of Dysbindin. We initially identified Dysbindin as an interaction partner of ID-specific Protein Myozap, both involved in Rho-dependent SRF-signaling [11]. Dysbindin is also a known constituent of biogenesis of lysosome-related organelles complex 1 (BLOC-1), which is associated with activities like endosomal-lysosomal regulation, protein sorting, etc. [25]. Interestingly, although the transcript level of Myozap was unaltered, Myozap was dramatically reduced at the protein level in Dysbindin-deficient mice, independent of surgery performed (TAC/Sham) or treatment given (PE/PBS) (Figure 4A–C and Figure S3A–C). Similarly, both Muted and Pallidin BLOC-1 members were strongly downregulated in *Dtnbp1*_KO mice at protein levels (Figure 4D–F), but not at transcript levels (Figure S3D,E). Overexpression of Dysbindin in NRVCMs however did not affect Pallidin or Myozap levels (Figure 4F–H). Overall, these data strongly suggest the importance of availability of Dysbindin in maintaining the integrity of its interaction partners.

## 4. Discussion

Dysbindin, the name originating from ‘dystobrevin binding protein 1′ was first discovered via yeast two-hybrid screening as a coiled-coil-domain containing protein that interacts with multiple dystrobrevins in muscle and brain [25]. Dysbindin is also a part of the BLOC-1 complex, where it mediates organogenesis and is required for directing protein cargos into the vesicle assembly that can reach nerve endings [26]. Dysbindin here plays a part in both pre- (impact glutamate synaptic function) and post-synaptic (works as a receptor component of postsynaptic density) neuronal transport [26]. In cardiomyocytes, we earlier found that Dysbindin interacts with the intercalated disc (ID) protein Myozap and strongly activates RhoA-mediated SRF-signaling in NRVCMs resulting in significant hypertrophy [11]. Conversely, knockdown of Dysbindin was effective in attenuating PE-induced cardiomyocyte hypertrophy [11]. Here, however, we found that these anti-hypertrophic effects were not translated in vivo where Dysbindin knockout mice exhibited an indifferent phenotype compared to that of wildtype counterparts after pressure overload due to transverse aortic constriction (TAC) or PE treatment. However, a striking finding was the strong downregulation of Myozap, Muted and Pallidin, direct interaction partners of Dysbindin, which implies that Dysbindin is required for the stability and proper stoichiometry of these protein complexes.

Pharmacologically induced alpha-adrenergic stimulation using PE or biomechanical stressors are inducers of cardiac hypertrophy and eventual disease-causing phenotypes. Our previous in vitro data suggested inhibitory effects of Dysbindin against cardiomyocyte hypertrophy induced due to either PE or ET in NRVCMs [11]. To study the translation of these in vitro effects in vivo, we employed pressure overload due to TAC- and PE-mediated hypertrophy induction in mice to assess if the absence of Dysbindin protects the heart from pathological cardiac hypertrophy in vivo. In contrast to our anticipation, the lack of Dysbindin did neither protect nor exaggerate cardiac hypertrophy. These data suggest that compensatory pathways must be at work in vivo, that are not active or inducible in vitro. Another important consideration is that the mouse model leads to constitutive deficiency throughout embryonic development. Future studies will have to show whether, e.g., an inducible knockout in adult mice will yield similar results.

Interestingly, though, a classical marker for cardiac contractility and emerging therapeutic target against heart failure, SERC2A [27,28,29,30,31,32], was significantly downregulated in Dysbindin deficient mice. Furthermore, Dysbindin deficiency strongly reduced the activation of ERK1/2, both in TAC operated and PE treated mice. Interestingly, it is established that the activation, i.e., phosphorylation of ERK1/2 is involved in the induction of prohypertrophic stimuli, lack of its activation also exhibits similar effects [26]. We thus believe that this downregulation of SERCA2A and pERK1/2 in *Dtnbp1*_KO mice might contribute, at least partly, to the observed cardiac remodeling after TAC or PE treatment.

The most prominent disease pathogenesis role of Dysbindin so far comes in schizophrenia via its interaction with Muted [33], where it modulates dopamine D2 receptor internalization and signaling [18,19,34,35]. Since the first reports describing dysbindin as a susceptibility protein for Schizophrenia it has now recently been investigated as a target for antipsychotic drug treatment [36,37]. On the other hand, various drugs like Clozapine, Quetiapine and Lovastatin, which are widely prescribed for antipsychotic medications have a possible association with cardiac diseases like electrocardiographic abnormalities and prolongation of QTc interval [38,39], suggesting an indirect role of systemic Dysbindin deficiency in drug-associated cardiac pathogenesis. These speculations however need experimental validations.

Interestingly, we observed that two of the members of this BLOC-1 complex were found to be downregulated in the heart with the knockout of Dysbindin. As all three, Dysbindin, Muted and Pallidin, are part of different sub-complexes, downregulation of the latter two along with Dysbindin points towards complete downregulation of both BLOC-1 complex and associated functions in the heart. BLOC-1 facilitates the protein trafficking on endosomes via interactions with BLOC-2, BLOC-3, BORC and AP-3 complexes [12,40,41,42]. The lack of functional consequences in Dysbindin-deficient mice is likely due to possible compensation to some extent by other complexes performing similar functions.

Similar to Muted and Pallidin, another cardiac binding partner of Dysbindin, Myozap, was dramatically reduced in *Dtnbp1*_KO mice. Like Dysbindin, we previously found that Myozap deficiency does not affect cardiac structure or function at baseline [43]. After TAC however, Myozap-deficiency led to accelerated cardiac hypertrophy, severe reduction of contractile function, signs of heart failure, and increased mortality. Mechanistically, reduced levels of Myozap might act in inducing cardiac pathology observed in *Dtnbp1*_KO mice. Taken together, the present study indicates that Dysbindin has a complex role in maintaining the stability and integrity of its interaction partners in forming functional complexes.

Limitations: While we did not observe a dramatic phenotype in mice lacking Dysbindin, either at baseline or upon stress, we cannot exclude the possibility that more sustained stressors e.g., prolonged TAC or PE treatment and/or other disease conditions (e.g., myocardial infarction) might reveal adverse consequences of Dysbindin deficiency. Furthermore, impact of Dysbindin deficiency on cardiomyocyte hypertrophy or death, if any, triggering observed fibrotic response after TAC or PE treatment was not accounted for in this study. Thus, further studies are needed to get deeper insights into if and how Dysbindin is involved in these processes.

## 5. Summary

The protein Dysbindin has been widely studied in the context of the pathogenesis of schizophrenia, where its downregulation is a cause for the disease. Some years ago, our group established its role in neonatal heart cells, where it induced pathogenic hypertrophy. The aim of the current study was to confirm that observation in various mice experiments, to find whether Dysbindin deficiency reduces cardiac hypertrophy. Although Dysbindin deficiency did not alter cardiac pathology upon cardiac stress, we observed dramatic reduction of some its interaction partners like Myozap, Muted and Pallidin, suggesting its important role in the stability of those proteins.

## Supplementary Materials

The following are available online at https://www.mdpi.com/2073-4409/9/11/2390/s1, Figure S1: (A) Agarose gel image depicting successful knockout of Dtnbp1 gene in the Dtnbp1_KO mice. (B) Immunoblot indicating the absence of Dysbindin protein in Dtnbp1_ko mice. (C) Transcript levels of SRF gene targets are not altered in Dysbindin-ko mice. Figure S2: Immunoblots indicating the increased levels of Dysbindin in the hearts of TAC operated (A) or PE treated (B) mice. (C) Percentage of mice survival post four weeks of operations. 8-week old wild-type (WT) and Dtnbp1_KO mice underwent TAC or Sham operations. *n* = 7 (WT-SHAM), 8 (WT-TAC), 8 (KO-SHAM), 8 (KO-TAC). Figure S3: (A) Immunoblots depicting Myozap protein levels in PBS/PE treated mice, its densitometric analysis against Tubulin shown in (B) and transcript levels in (C). Transcript levels of Pallidin (D) and Muted (E) are shown in a bar graph in Dysbindin-KO mice compared to wild-type littermates upon TAC. (F) Immunoblots depicting Dysbindin, Pallidin and Myozap levels in NRVCMs overexpressing LacZ (control) or Dysbindin, densitometric analysis of which for Myozap and Pallidin against GAPDH are shown in (G) and (H), respectively. Statistical significance was calculated by two-way ANOVA. *Error bars* show mean ± S.E. **, *p* < 0.01; ***, *p* < 0.001. Table S1: Raw data for the immunoblot presented in Figure 2L. Table S2: Raw data for the immunoblot presented in Figure 2N. Table S3: Raw data for the immunoblot presented in Figure 3L. Table S4: Raw data for the immunoblot presented in Figure 3N. Table S5: Raw data for the immunoblot presented in Figure 4A. Table S6: Raw data for the immunoblot presented in Figure 4D for MUTED. Table S7: Raw data for the immunoblot presented in Figure 4D for Pallidin. Table S8: Raw data for the immunoblot presented in Supplementary Figure S3A. Table S9: Raw data for the immunoblot presented in Supplementary Figure S3F for Myozap. Table S10: Raw data for the immunoblot presented in Supplementary Figure S3F for Pallidin.
