# Supplementary material for: Dysbindin deficiency Alters Cardiac BLOC-1 Complex and Myozap Levels in Mice
Source: Cells. 2020 Oct 31;9(11):2390. doi: 10.3390/cells9112390 (PMC7692170; doi:10.3390/cells9112390)
Supplement: Supplementary file 1 [file cells-09-02390-s001.pdf]

Dysbindin-deficiency alters cardiac BLOC-1 complex and Myozap levels in mice

Ankush Borlepawar [1,2], Nesrin Schmiedel [1,2], Matthias Eden [1,2], Lynn Christen [1], Alexandra Rosskopf [1,2], Derk Frank [1,2], Renate Lüllmann-Rauch [3], Norbert Frey [1,2] and Ashraf Yusuf Rangrez [1,2,*]

1 Department of Internal Medicine III (Cardiology, Angiology, Intensive Care), University Medical Center Kiel, 24105 Kiel, Germany; ankush.borlepawar@uksh.de (A.B.); nesrin.schmiedel@uksh.de (N.S.); matthias.eden@uksh.de (M.E.); lynn.christen@web.de (L.C.); alexandra.rosskopf@uksh.de (A.R.); derk.frank@uksh.de (D.F.); norbert.frey@uksh.de (N.F.)
2 DZHK (German Centre for Cardiovascular Research), partner site Hamburg/Kiel/Lübeck, 24105 Kiel, Germany
3 Institute of Anatomy, Christian-Albrechts-University Kiel, 24118 Kiel, Germany; r.lullmann@anat.uni-kiel.de
* Correspondence: ashraf.rangrez@uksh.de; Tel.: +49-431-500-22966; Fax: +49-431-500-22938

Supplementary Figures

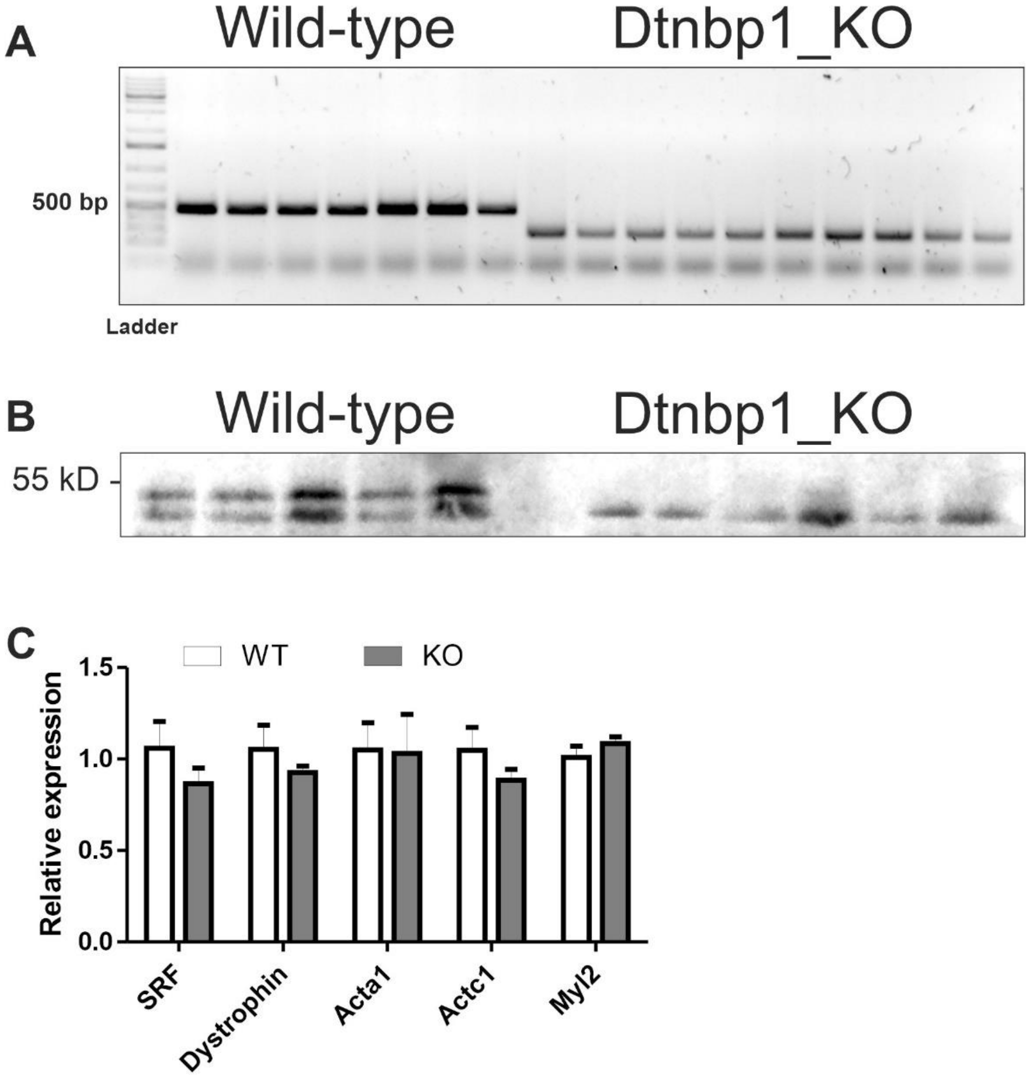

Figure S1. (**A**) Agarose gel image depicting successful knockout of Dtnbp1 gene in the Dtnbp1_KO mice. (**B**) Immunoblot indicating the absence of Dysbindin protein in Dtnbp1_ko mice. (**C**) Transcript levels of SRF gene targets are not altered in Dysbindin-ko mice.

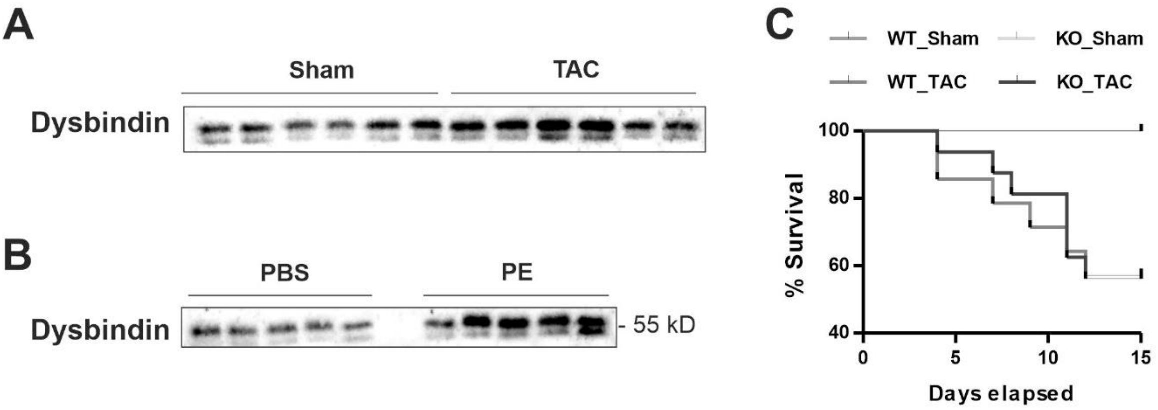

Figure S2. Immunoblots indicating the increased levels of Dysbindin in the hearts of TAC operated (**A**) or PE treated (**B**) mice. (**C**) Percentage of mice survival post four weeks of operations. 8-week old wild-type (WT) and Dtnbp1_KO mice underwent TAC or Sham operations. $n = 7$ (WT-SHAM), 8 (WT-TAC), 8 (KO-SHAM), 8 (KO-TAC).

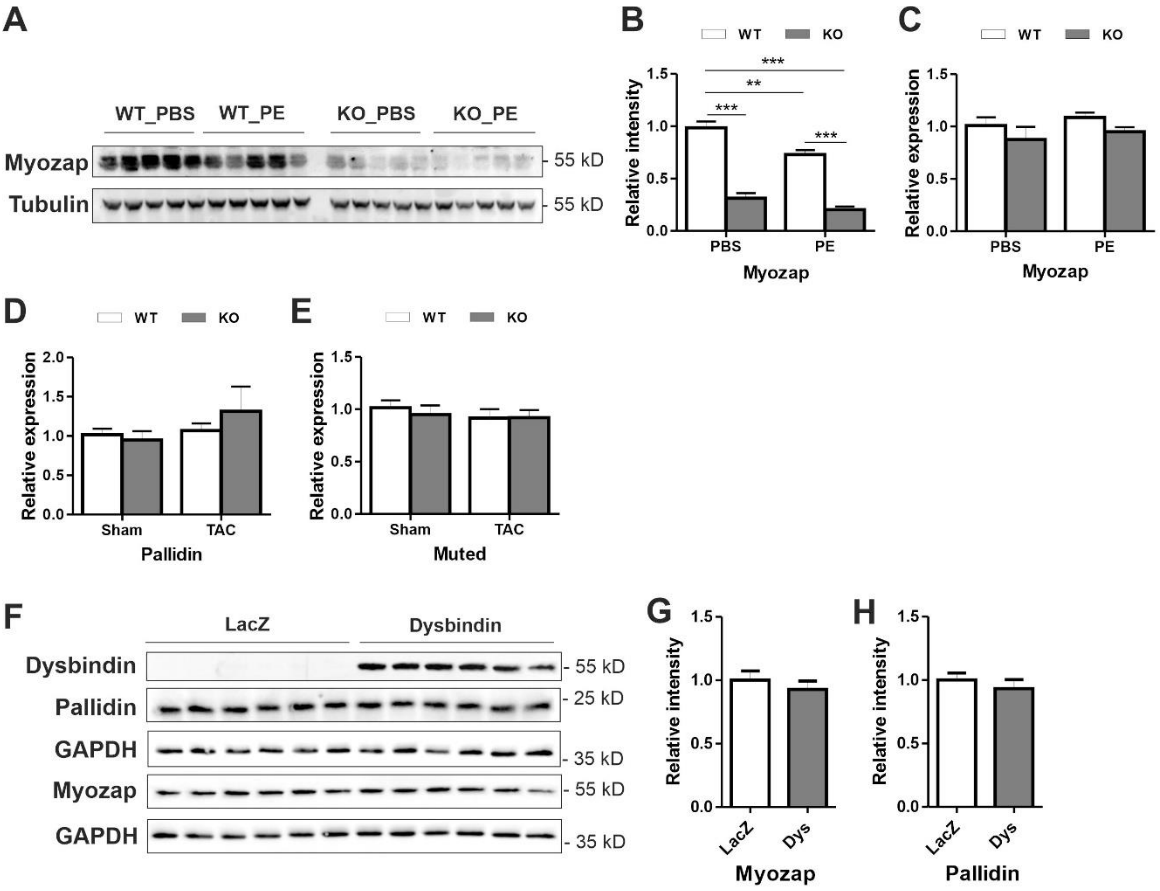

Figure S3. (**A**) Immunoblots depicting Myozap protein levels in PBS/PE treated mice, its densitometric analysis against Tubulin shown in (**B**) and transcript levels in (**C**). Transcript levels of Pallidin (**D**) and Muted (**E**) are shown in a bar graph in Dysbindin-KO mice compared to wild-type littermates upon TAC. (**F**) Immunoblots depicting Dysbindin, Pallidin and Myozap levels in NRVCMs overexpressing LacZ (control) or Dysbindin, densitometric analysis of which for Myozap and Pallidin against GAPDH are shown in (**G**) and (**H**), respectively. Statistical significance was calculated by two-way ANOVA. *Error bars* show mean ± S.E. **, $p < 0.01$; ***, $p < 0.001$.

Supplementary Tables

Table S1. Raw data for the immunoblot presented in Figure 2L.

WT_Sham	WT_TAC	KO_Sham	KO_TAC
1.130415	0.599836	0.929451	0.75464
1.327263	0.350664	0.563498	0.683463
1.102144	0.909246	0.509753	0.584217
0.4139448	0.764626	0.881415	1.011252
0.8673782	0.701512	0.56093	0.699081
1.098796	0.703609	0.633638	0.412821
1.060059	0.70555	0.834172	0.622075
	0.567467		0.334144
	0.422139		

Table S2. Raw data for the immunoblot presented in Figure 2N.

WT_Sham	WT_TAC	KO_Sham	KO_TAC
0.981628	0.529585	0.835823	0.381385
0.914605	0.689421	0.788225	0.491211
0.659251	0.359446	0.575456	0.438407
0.504967	0.416684	0.735265	0.572974
1.921726	0.519285	0.849885	0.650203
1.213576	0.86997	0.738938	0.580168
0.804247	1.047373		0.529709
	1.195542		0.322031
	0.886042		0.32278

Table S3. Raw data for the immunoblot presented in Figure 3L.

WT_PBS	WT_PE	KO_PBS	KO_PE
1.032549	1.063999	0.923573	0.801004
0.8893051	0.841239	1.310846	1.191159
0.9578611	0.760589	0.930995	1.134456
0.9261611	0.513888	1.209022	0.864042
1.194124	0.61727	0.823035	0.857778

Table S4. Raw data for the immunoblot presented in Figure 3N.

WT_PBS	WT_PE	KO_PBS	KO_PE
0.952363	0.677961	0.889158	0.811422
0.881515	0.661783	0.823069	0.573356
1.1925	0.640403	0.470239	0.517891
1.137246	0.788093	0.378706	0.746602
0.836376	0.567026	0.672541	0.891102

Table S5. Raw data for the immunoblot presented in Figure 4A.

WT_Sham	WT_TAC	KO_Sham	KO_TAC
1.318202	1.055972	1.139635	1.070713
1.147561	0.985257	1.123945	0.938593
1.336603	0.794749	1.108471	1.048678
0.2888859	0.938593	1.238481	0.958315
1.622897	1.139635	1.282156	0.99901
0.6410778	1.238481	1.282156	1.196294
1.645552	1.4425	1.503757	0.828499
	1.247095		0.978451
	1.355262		

Table S6. Raw data for the immunoblot presented in Figure 4D for MUTED.

WT_Sham	WT_TAC	KO_Sham	KO_TAC
0.941335	0.532949	0.562344	0.418654
1.101041	0.712813	0.594946	0.513925
0.9576236	0.675247	0.676188	0.391747
0.8323443	0.643655	0.626808	0.295592
1.129014	0.829598	0.459404	0.177729
1.266364	0.741624	0.674028	0.220474
0.7722768	0.814755	0.117813	0.528019
	0.945918		0.52887
	0.848501		

Table S7. Raw data for the immunoblot presented in Figure 4D for Pallidin.

WT_Sham	WT_TAC	KO_Sham	KO_TAC
1.137589	0.921732	0.104549	0.126833
0.7280808	0.764823	0.080059	0.106126
1.13433	1.381596	0.03916	0.102144
0.9087197	0.905451	0.072325	0.136754
1.193995	0.778779	0.387723	0.230536
0.8647355	0.709182	0.380938	0.246163
1.03255	0.988916	0.054611	0.301183
	0.987928		0.247043
	0.801107		

Table S8. Raw data for the immunoblot presented in Supplementary Figure 3A.

WT_PBS	WT_PE	KO_PBS	KO_PE
0.8228166	0.75231	0.47453	0.1675
0.982429	0.61378	0.34074	0.1275
1.179879	0.84115	0.2078	0.16913
1.014876	0.78883	0.33053	0.27298
0.929543	0.64944	0.20687	0.27946

Table S9. Raw data for the immunoblot presented in Supplementary Figure 3F for Myozap.

LacZ	Dys
0.802124	0.924469
1.014541	0.877002
1.278518	1.179984
1.038124	0.968263
1.060372	0.938533
0.806321	0.683319

Table S10. Raw data for the immunoblot presented in Supplementary Figure 3F for Pallidin.

LacZ	Dys
0.9239052	1.115758
1.071879	0.7488091
1.146348	1.167691
0.9513502	0.9155022
1.115312	0.8753819
0.7912063	0.7731408
